# Complete Genomic Sequence of an Avian Pathogenic *Escherichia coli* Strain of Serotype O7:HNT

**DOI:** 10.1128/genomeA.01611-15

**Published:** 2016-01-28

**Authors:** Renato P. Maluta, Bryon Nicholson, Catherine M. Logue, Lisa K. Nolan, Thaís C. G. Rojas, Wanderley Dias da Silveira

**Affiliations:** a Department of Genetics, Evolution and Bioagents, Bacterial Molecular Biology Laboratory, Institute of Biology, State University of Campinas (UNICAMP), Campinas, SP, Brazil; b Department of Veterinary Microbiology and Preventive Medicine, College of Veterinary Medicine, Iowa State University, Ames, Iowa, USA

## Abstract

Avian pathogenic *Escherichia coli* (APEC) is associated with colibacillosis in poultry. Here, we present the first complete sequence of an APEC strain of the O7:HNT serotype and ST73 sequence type, isolated from a broiler with cellulitis. Complete genomes of APEC with distinct genetic backgrounds may be useful for comparative analysis.

## GENOME ANNOUNCEMENT

*Escherichia coli* is a versatile bacterium exhibiting a high degree of genomic plasticity (1). Most strains are harmless; however, a subset of *E. coli* cause intestinal (intestinal pathogenic *E. coli* [InPEC]) or extraintestinal (extraintestinal pathogenic *E. coli* [ExPEC]) diseases in humans and animals (2). In mammals, extraintestinal diseases caused by ExPEC include neonatal meningitis, urinary tract infections, septicemia, mastitis, and pyometra (3–6). ExPEC that produces disease in multiple avian species is known as avian pathogenic *E. coli* (APEC) (7). Collectively, these diseases are known as colibacillosis (7). Colibacillosis is responsible for remarkable losses in the poultry industry worldwide (7). Besides the detrimental impact of colibacillosis on poultry health and the economic viability of the poultry industry, there is evidence that ExPEC from animal origin might contaminate food and cause disease in human beings (8).

A number of APEC have been sequenced. Among them, there are strains belonging to the serogroups O1 (9), O2 (10), and O78 (11), and also nontypeable strains (12). Serogroup O7 strains are commonly detected in human ExPEC (13), where they are known to cause urinary tract infections (13), neonatal meningitis (3), and septicemia (14), and may also occur in APEC (15), where they have caused avian cellulitis in broilers (4) and colibacillosis in turkeys and chickens (16). Most O7 sequenced genomes represent human ExPEC strains, such as uropathogenic *E. coli* (UPEC) IAI39 (accession no. CU928164), UPEC UMN026 (17), and neonatal meningitis *E. coli* (NMEC) CE10 (18). To our knowledge, a complete genome with an APEC O7 strain has not been reported.

The plasmidless *E. coli* strain RS76 of the serotype O7:HNT and sequence type ST93 was isolated from the carcass of a slaughtered broiler diagnosed with avian cellulitis in Brazil (4). The genome was sequenced using 150-bp Ilumina paired-end (15,697,322 reads) and 100-bp mate pair (24,920,310 reads) libraries. *De novo* assembly was performed with SPAdes 3.0 (19). The assembly presented an average coverage of 200×. The resulting contigs and scaffolds were ordered with progressiveMauve (20) using *E. coli* BL21(DE3) (accession no. AM946981) as a reference. Gaps were eliminated with PCR and subsequent Sanger sequencing. The APEC RS76 genome sequence was annotated by the NCBI Prokaryotic Genomes Automatic Annotation Pipeline (PGAAP). The consistency of the PGAAP annotation was verified against a previous Prokka 1.11 annotation (21).

The single chromosome presented two gaps with unknown length. The total length, excluding gaps, was 4,689,208 bp (50.7% GC content). This chromosome presented 4,407 coding sequences, 84 tRNA encoding genes, and 7 rRNA encoding operons.

There are two general issues regarding the impact of APEC that still need to be elucidated. The genetic determinants associated with APEC pathogenicity in avian species are not fully understood yet. Also, the role of APEC as a zoonotic agent is controversial. The sequencing of complete APEC genomes presenting distinct genetic backgrounds may be useful for future comparative genomic analysis addressing those issues.

### Nucleotide sequence accession number.

The complete sequence of strain RS76 was deposited in GenBank under the accession number CP013048.
